# Metformin overdose, but not lactic acidosis *per se*, inhibits oxygen consumption in pigs

**DOI:** 10.1186/cc11332

**Published:** 2012-05-08

**Authors:** Alessandro Protti, Francesco Fortunato, Massimo Monti, Sarah Vecchio, Stefano Gatti, Giacomo P Comi, Rachele De Giuseppe, Luciano Gattinoni

**Affiliations:** 1 Dipartimento di Anestesia, Rianimazione (Intensiva e Sub-Intensiva) e Terapia del Dolore, Fondazione IRCCS Ca' Granda - Ospedale Maggiore Policlinico, Università degli Studi di Milano, Via Francesco Sforza 35, Milano 20122, Italy; 2 Centro Dino Ferrari - Dipartimento di Scienze Neurologiche, Fondazione IRCCS Ca' Granda - Ospedale Maggiore Policlinico, Università degli Studi di Milano, Via Francesco Sforza 35, Milano 20122, Italy; 3 Centro Nazionale di Informazione Tossicologica - Centro Antiveleni, Fondazione IRCCS Salvatore Maugeri, Via Salvatore Maugeri 10, Pavia 27100, Italy; 4 Centro di Ricerche Chirurgiche Precliniche, Fondazione IRCCS Ca' Granda - Ospedale Maggiore Policlinico, Università degli Studi di Milano, Via Francesco Sforza 35, Milano 20122, Italy; 5 Fondazione Fratelli Confalonieri, Dipartimento di Scienze Mediche, Università degli Studi di Milano, Fondazione IRCCS Ca' Granda - Ospedale Maggiore Policlinico, Via Francesco Sforza 35, Milano 20122, Italy

## Abstract

**Introduction:**

Hepatic mitochondrial dysfunction may play a critical role in the pathogenesis of metformin-induced lactic acidosis. However, patients with severe metformin intoxication may have a 30 to 60% decrease in their global oxygen consumption, as for generalized inhibition of mitochondrial respiration. We developed a pig model of severe metformin intoxication to validate this clinical finding and assess mitochondrial function in liver and other tissues.

**Methods:**

Twenty healthy pigs were sedated and mechanically ventilated. Ten were infused with a large dose of metformin (4 to 8 g) and five were not (sham controls). Five others were infused with lactic acid to clarify whether lactic acidosis *per se *diminishes global oxygen use. Arterial pH, lactatemia, global oxygen consumption (VO_2_) (metabolic module) and delivery (DO_2_) (cardiac output by thermodilution) were monitored for nine hours. Oxygen extraction was computed as VO_2_/DO_2_. Activities of the main components of the mitochondrial respiratory chain (complex I, II and III, and IV) were measured with spectrophotometry (and expressed relative to citrate synthase activity) in heart, kidney, liver, skeletal muscle and platelets taken at the end of the study.

**Results:**

Pigs infused with metformin (6 ± 2 g; final serum drug level 77 ± 45 mg/L) progressively developed lactic acidosis (final arterial pH 6.93 ± 0.24 and lactate 18 ± 7 mmol/L, *P *< 0.001 for both). Their VO_2 _declined over time (from 115 ± 34 to 71 ± 30 ml/min, *P *< 0.001) despite grossly preserved DO_2 _(from 269 ± 68 to 239 ± 51 ml/min, *P *= 0.58). Oxygen extraction accordingly fell from 43 ± 10 to 30 ± 10% (*P *= 0.008). None of these changes occurred in either sham controls or pigs infused with lactic acid (final arterial pH 6.86 ± 0.16 and lactate 22 ± 3 mmol/L). Metformin intoxication was associated with inhibition of complex I in the liver (*P *< 0.001), heart (*P *< 0.001), kidney (*P *= 0.003), skeletal muscle (*P *= 0.012) and platelets (*P *= 0.053). The activity of complex II and III diminished in the liver (*P *< 0.001), heart (*P *< 0.001) and kidney (*P *< 0.005) while that of complex IV declined in the heart (*P *< 0.001).

**Conclusions:**

Metformin intoxication induces lactic acidosis, inhibits global oxygen consumption and causes mitochondrial dysfunction in liver and other tissues. Lactic acidosis *per se *does not decrease whole-body respiration.

## Introduction

Metformin is the drug of choice for adults with type 2 diabetes [1]. It is a safe drug [2] although lactic acidosis rarely develops as a side effect [3-5]. In most of the cases, drug use is only coincidental and lactic acidosis is due to concomitant hypoxia, tissue hypoperfusion or liver failure (metformin-associated lactic acidosis) [6]. Less frequently, no other major risk factor can be identified and drug accumulation, prompted by renal failure for instance, is the most probable cause of the syndrome (metformin-induced lactic acidosis) [7].

The pathogenesis of metformin-induced lactic acidosis remains unclear. Some authors even question whether metformin *per se *can cause lactic acidosis [8]. Some others mainly attribute it to changes in liver lactate metabolism [9]. Metformin readily accumulates in hepatocytes, that express the organic cation transporter 1 [10], and inhibits their mitochondrial respiration in a dose-dependent manner [11-13]. As a possible result, liver lactate output increases [11-13] and uptake decreases (along with gluconeogenesis) [12,14]. However, critically ill metformin-intoxicated patients may have a 30 to 60% decrease in their global oxygen extraction and consumption [7], as for generalized (and not solely hepatic) mitochondrial dysfunction. Lactate overproduction from tissues other than the liver may then occur [15].

The aim of the present study was to clarify whether metformin intoxication inhibits whole-body respiration and alters mitochondrial function in the liver and other tissues.

## Materials and methods

The study complied with international recommendations [16] and was approved by the Italian Ministry of Health (protocol number 212).

### Surgical preparation

Twenty healthy pigs (22 ± 2 kg) were sedated with tiletamine/zolazepam (5 mg/kg intramuscular (IM)) and medetomidine hydrochloride (0.025 mg/kg IM) and infused with ceftriaxone (1 g intravenous (IV)), propofol (50 mg IV) and tramadol (50 mg IV). Tracheotomy and cystostomy were performed and carotid artery, internal jugular vein and pulmonary artery were cannulated. Thereafter, mechanical ventilation (oxygen inspiratory fraction of 0.5) and infusion of propofol (80 to 100 mg/hour), medetomidine (50 μg/hour), pancuronium bromide (8 to 10 mg/hour) and saline (50 ml/hour) were kept constant until end of the experiment.

### Study design

After six hours of stabilization and baseline recordings (time 0), ten pigs were infused with 4 to 8 g of metformin hydrochloride (Sigma-Aldrich; St. Louis, MO, USA) (1.6 g in 20 ml of saline per hour, over 2.5 to 5 hours) and five were not (sham controls). Five other pigs were continuously infused with lactic acid (30% in water) (Sigma-Aldrich) to clarify whether lactic acidosis (that always developed in animals treated with metformin) directly inhibits global oxygen consumption. Lactic acid infusion was hourly adjusted to mimic the rise in lactatemia observed in metformin-intoxicated pigs.

Data collection always lasted nine hours (animals were then sacrificed). Arterial and mixed venous blood gases, lactatemia and glycaemia were measured every hour. Oxygen delivery (DO_2_) was calculated as: 13.6*CO*Hb*SaO_2_, where CO, Hb and SaO_2 _are cardiac output (thermodilution), arterial haemoglobin concentration and oxygen saturation. Oxygen consumption (VO_2_) was measured with a COVX metabolic module (GE Healthcare; Madison, WI, USA) and extraction computed as VO_2_/DO_2_. Water balance was calculated as the difference between overall saline input and urinary output. In pigs infused with metformin, final (time nine) serum drug concentration was measured with high performance liquid chromatography.

Hypotension (mean arterial pressure < 60 mmHg) was always treated with additional saline and norepinephrine, hypoglycaemia (< 60 mg/dl) with glucose and hypothermia (body core temperature < 37°C) with active warming.

Further investigations were only performed in pigs infused with metformin and sham controls.

### Mitochondrial function tests

Before sacrifice, ethylenediaminetetraacetic acid-anticoagulated blood was collected and sedimented for 45 minutes at 4°C. The top three-quarters of platelet-rich plasma were washed with distilled water, centrifuged at 5000 g for 10 minutes (14500 g from the second cycle on) and washed with phosphate-buffered saline until a clear platelet pellet could be stored at -80°C (two to three cycles were usually required). After sacrifice (KCl 40 mEq IV), samples of heart, liver, kidney and skeletal muscle were immediately stored in liquid nitrogen.

At the time of analysis, platelet pellet was diluted in buffer (300 to 400 μl) (KCl 120 mM, ethyl piperazine ethane sulfonic acid 20 mM, MgCl_2 _5 mM and ethylene glycol tetraacetic acid 1 mM; pH 7.2), sonicated (two cycles at 60 W for 10 seconds) and then centrifuged (750 g for 10 minutes) while kept at 4°C. Tissue fragments were diluted (1:10) in the same buffer, homogenized (three cycles at 350 g for one minute) and centrifuged as above. We measured the activity of respiratory chain nicotinamide adenine dinucleotide-ubiquinone 1 reductase (complex I), succinate-cytochrome c reductase (complex II + III) and cytochrome c oxidase (complex IV) on supernatants, using spectrophotometry (at 30°C). Results are expressed relative to citrate synthase activity, a marker of mitochondrial content [17]. Proteins were measured with Lowry's method.

### Lactate-to-pyruvate ratio

At the end of the study, blood was withdrawn from six intoxicated and five control animals into heparinized tubes and immediately centrifuged (2500 g for 10 minutes). Plasma was then stored at -80°C. Lactate and pyruvate levels were measured on perchloric acid-deproteinezed plasma using high-performance liquid chromatography. Final lactate-to-pyruvate ratios were calculated.

### Oxidative stress

Final serum reactive oxygen species and total antioxidant capacity were measured with d-ROMs and Oxy-adsorbent tests (Diacron International; Grosseto, Italy) [18]. These assays are based on spectrophotometric quantification of a color compound that forms in response to oxidation. The d-ROMs test evaluates the concentration of reactive oxygen metabolites, namely hydroperoxides. Results are expressed in arbitrary units, called U Carr (one U Carr corresponds to 0.08 mg/dl of hydrogen peroxide). The Oxy-adsorbent test measures the overall capacity of a biological sample to absorb exogenous hypochlorous acid (HClO). Results are expressed as the maximum concentration of hypochlorous acid that can be absorbed by the sample.

### Statistical analysis

Data are reported as mean and standard deviation (SD). Based on distribution (Shapiro-Wilk test), we used Student's *t *or Wilcoxon rank sum tests to compare two groups. Interactions between groups and time were tested with two-way repeated measures analysis of variance. Non-normally distributed data were firstly transformed in ranks [19]. Unless otherwise stated, *P *values reported in brackets refer to analysis of these interactions. *Post hoc *comparisons were performed with Holm-Sidak method. Correlations were tested with linear regression analysis (R^2^). A *P *value < 0.05 was considered statistically significant (SigmaPlot version 11.0, Jandel Scientific Software; San Jose, CA, USA).

## Results

**Figure 1:**
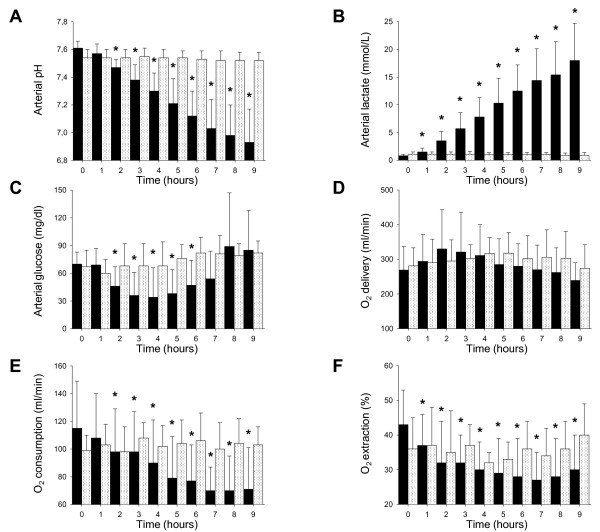
**Major metabolic changes during metformin infusion**. Ten pigs were infused with metformin (black bars) and five pigs were not (white dotted bars). Infusion of metformin started after baseline recordings (time 0) and lasted 2.5 to 5 hours (dose infused ranged between 4 and 8 g). Arterial pH **(A)**, lactatemia **(B)**, glycemia **(C)**, global oxygen (O_2_) delivery **(D)**, consumption **(E) **and extraction **(F) **were recorded every hour. Data are reported as mean and SD. *P < 0.05 vs. time 0 within group. To convert glucose in mmol/L, divide by 18.

All animals survived until end of the study. In sham controls, none of the variables of interest changed over time (Figure 1, Additional file 1). Conversely, pigs infused with metformin (6 ± 2 g; final serum drug concentration 77 ± 45 (range 3 to 146) mg/L) had, on average, severe lactic acidosis (final arterial pH 6.93 ± 0.24 (range 6.80 to 7.57); lactate 18 ± 7 (range 1 to 24) mmol/L) (Figure 1). Arterial pH decreased and lactatemia increased proportionally to final serum metformin level (R^2 ^0.54, *P *= 0.015 and R^2 ^0.40, *P *= 0.049, respectively). Hypoglycaemia usually developed (*P *= 0.002): more glucose was infused, on average, in intoxicated than in control animals (15 ± 7 vs. 3 ± 5 ml/hour of a 33% solution; *P *= 0.003).

Metformin intoxication progressively inhibited global oxygen extraction (*P *= 0.008) and consumption (*P *< 0.001) and increased mixed venous oxygen saturation (from 65 ± 6 up to 78 ± 7%, *P *< 0.001), despite no change in oxygen delivery (*P *= 0.58) (Figure 1). The overall rise in blood lactate levels was proportional to the decrease in global oxygen use (R^2 ^0.70, *P *= 0.002). Body temperature fell from 38.0 ± 2.0 to 36.6 ± 1.2°C (*P *< 0.001).

Hemodynamic changes are shown in Additional file 1. Pigs infused with metformin became hypotensive (*P *< 0.001) and therefore received much more fluids (final water balance 1172 ± 834 vs. -139 ± 242 ml, *P *= 0.011) and norepinephrine (< 0.001) than controls. Cardiac output (not measured in one case) never significantly changed (*P *= 1.0).

**Figure 2:**
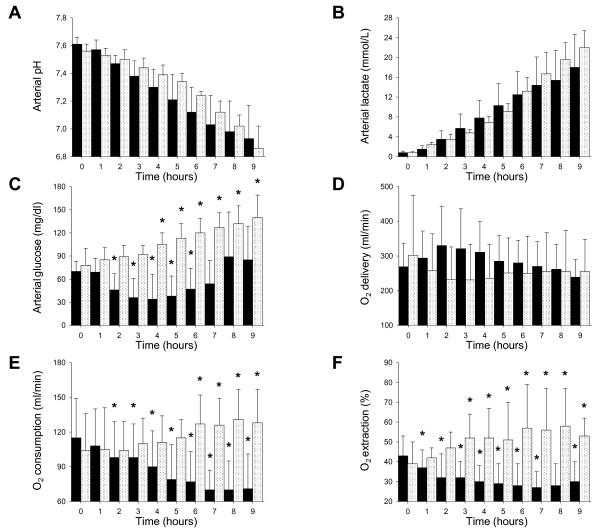
**Major metabolic changes during metformin or lactic acid infusion**. Ten pigs were infused with metformin (black bars) and five pigs with lactic acid (white dotted bars). Infusion of metformin started after baseline recordings (time 0) and lasted 2.5 to 5 hours (dose infused ranged between 4 and 8 g). Infusion of lactic acid started after baseline recordings (time 0) and continued until end of the study, with velocity adjusted to mimic the rise of lactatemia of metformin-intoxicated pigs. Arterial pH **(A)**, lactatemia **(B)**, glycemia **(C)**, global oxygen (O_2_) delivery **(D)**, consumption **(E) **and extraction **(F) **were recorded every hour. Data are reported as mean and SD. *P < 0.05 vs. time 0 within group. To convert glucose in mmol/L, divide by 18.

Infusion of lactic acid decreased arterial pH (down to 6.86 ± 0.16) and increased lactatemia (up to 22 ± 3 mmol/L) just as metformin did. Nonetheless, global oxygen use and extraction augmented (*P *< 0.001 for both), mixed venous oxygen saturation diminished (from 65 ± 11 down to 39 ± 14%, *P *< 0.001) and delivery slightly, spontaneously, increased (*P *= 0.017) (Figure 2). Hemodynamic changes are reported in Additional file 2.

### Mitochondrial function tests

**Figure 3:**
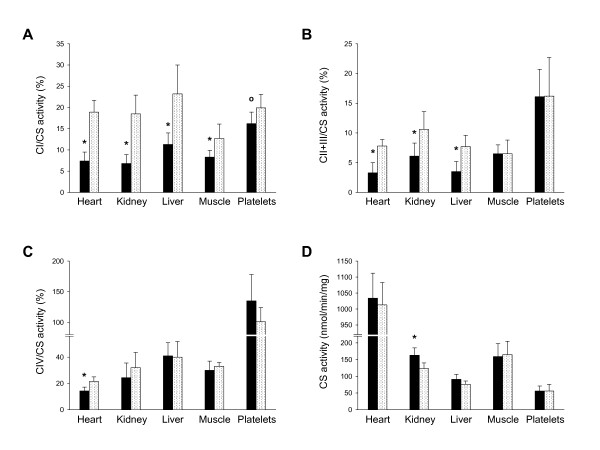
**Mitochondrial changes following metformin intoxication**. Ten pigs were infused with metformin (black bars) and five pigs were not (white dotted bars). Activities of complex I (CI) **(A)**, complex II and III (CII+III) **(B) **complex IV (CIV) **(C) **and citrate synthase (CS) **(D) **were measured at the end of study in heart, kidney, liver, skeletal muscle and platelets. Data are reported as mean and SD. °*P *= 0.05 vs. saline. *P < 0.05 vs. saline. Platelet mitochondrial function was only measured in seven of the intoxicated pigs.

Relative to sham controls, respiratory chain enzymes were variably inhibited in pigs infused with metformin (Figure 3). Complex I activity was lower in liver (*P *< 0.001), heart (*P *< 0.001), kidney (*P *= 0.003), skeletal muscle (*P *= 0.012) and platelets (*P *= 0.053). The activity of complex II and III was inhibited in liver (*P *< 0.001), heart (*P *< 0.001) and kidney (*P *< 0.005) while that of complex IV was low in heart (*P *< 0.001). Citrate synthase activity slightly increased in kidney (*P *= 0.003) and liver (*P *= 0.066).

### Lactate-to-pyruvate ratio

Intoxicated pigs had final plasma pyruvate levels slightly higher (197 ± 52 vs. 121 ± 23 μmol/L, *P *= 0.08) and lactate-to-pyruvate ratios largely higher (122 ± 32 vs. 10 ± 2, *P *< 0.001) than controls.

### Oxidative stress

Final serum reactive oxygen species (590 ± 153 vs. 628 ± 85 U Carr, *P *= 0.632) and total antioxidant capacity (227 ± 64 vs. 218 ± 33 μmol HClO/ml, *P *= 0.767) did not differ between intoxicated and control animals.

## Discussion

This work demonstrates that metformin intoxication: 1) causes lactic acidosis; 2) inhibits oxygen consumption (while lactic acidosis *per se *does not) and 3) impairs mitochondrial function in liver and other tissues.

Lactic acidosis is commonly due to cellular hypoxia [20]. Whenever oxygen delivery diminishes relative to demand, extraction increases to maintain a normal aerobic (mitochondrial) energy production. When this compensatory mechanism is exhausted, anaerobic (extra-mitochondrial) glycolysis accelerates, lactate production increases [21] and acidosis develops. Lactate released from skeletal muscle into systemic circulation to be taken up as an energy substrate by distal organs may further increase blood lactate levels [22].

Lactic acidosis can also occur independently from cellular hypoxia [23,24], as in these experiments. In fact, following metformin infusion in healthy pigs, oxygen use declined and lactic acidosis arose only because oxygen extraction, but not delivery, diminished. Accordingly, mixed venous oxygen saturation increased, suggesting that delivery even exceeded demand. The decline in body temperature further indicates hypometabolism. These data confirm our previous clinical findings: patients with lactic acidosis due to metformin accumulation have abnormally low oxygen extraction and consumption, high venous oxygen tension and are usually hypothermic [7]. Severe metformin intoxication therefore resembles cyanide poisoning [25], when cells become unable to use otherwise available oxygen and energy production largely depends on glycolysis. The fact that oxygen consumption never diminished in animals infused with lactic acid (and no metformin) demonstrates that lactic acidosis *per se *cannot explain our present findings.

Serum metformin levels in intoxicated pigs (77 ± 45 mg/L) were similar to those we have previously recorded in humans (61 ± 25 mg/L; therapeutic level is < 4 mg/L) [7]. Severity of intoxication in pigs correlated with that of lactic acidosis. One single animal had a normal final serum drug concentration (possibly due to efficient drug urinary excretion) with only mild and transient metabolic alterations. All other pigs had clearly toxic serum drug levels with overt and persistent lactic acidosis.

Metformin variably accumulates in virtually every tissue [26]. *In vitro*, it dose-dependently inhibits mitochondrial function even in cells other than hepatocytes [27-32]. We have now shown that, *in vivo *and at toxic dose, it causes mitochondrial dysfunction not just in liver but also in heart, kidney, skeletal muscle and platelets (with differences in severity possibly explained by uneven drug distribution). Lower global oxygen use may thus reflect a generalized inhibition of mitochondrial respiration, which may lead to hepatic and extra-hepatic lactate overproduction. The large increase in blood lactate-to-pyruvate ratios may consistently signal accelerated glycolysis and lactate generation in face of diminished mitochondrial energy production [33].

Previous reports have shown that chronic therapeutic levels of metformin can stimulate mitochondrial biogenesis [34], the process by which cells form new mitochondria. Increased citrate synthase activity in kidney and liver is consistent with this finding. But still, in our present model of acute intoxication, mitochondrial function was mainly, largely, depressed. Other molecules variably modulate mitochondrial metabolism depending on dose and time of administration. For instance, low levels of nitric oxide chronically stimulate mitochondrial biogenesis [35] whereas toxic levels acutely inhibit respiratory chain enzymes [36].

How metformin affects mitochondrial function remains controversial [11,12] and our study did not specifically address this issue. It may increase the formation of reactive oxygen species [37] that may ultimately disrupt mitochondrial integrity [38]. However, we could not demonstrate any clear change in serum markers of oxidative stress following metformin intoxication. Mitochondrial dysfunction in platelets, that are anucleated cells, suggests that *de novo*, nuclear-encoded, messengers may not be required.

Some of the limitations of this study deserve a comment. First, we used healthy pigs whereas patients with accidental metformin intoxication typically suffer from diabetes, renal failure and other comorbidities. Doing so, we aimed at investigating the pure effect of drug accumulation on global oxygen use and metabolism. Second, pigs infused with metformin also received much more fluids and norepinephrine (to treat hypovolemia and systemic vasodilatation) and glucose (to correct hypoglycaemia) than controls. None of these other factors, however, can explain our present findings. In fact, respiratory chain enzymes activities were expressed relative to citrate synthase activity, to correct for any difference in mitochondrial density (possibly due to edema, for instance). Norepinephrine increases oxygen consumption *in vivo *[39] and does not grossly alter porcine hepatic and skeletal muscle mitochondrial respiration *in vitro *[40,41]. Although (intracellular) hypoglycaemia can diminish oxygen consumption, it can hardly explain lactate overproduction, since lactate is generated from glucose. Notably, changes in oxygen consumption and blood lactate levels persisted even when hypoglycaemia was reversed. Third, cellular respiration and lactate generation were not directly measured.

## Conclusions

The development of lactic acidosis during metformin intoxication is associated with diminished global oxygen consumption and clear evidence of mitochondrial dysfunction in liver and other tissues. Lactic acidosis *per se *does not inhibit whole-body respiration.

## Key messages

• Severe metformin intoxication largely decreases global oxygen consumption and impairs mitochondrial function in heart, kidney, liver, skeletal muscle and platelets of otherwise healthy pigs.

• Lactic acidosis *per se *does not decrease whole-body respiration.

• Diffuse inhibition of cellular respiration and secondary lactate overproduction may contribute to the development of metformin-induced lactic acidosis.

## Abbreviations

CO: cardiac output; DO_2_: global oxygen delivery; Hb: haemoglobin; HClO: hypochlorous acid; IM: intramuscular; IV: intravenous; O_2_: oxygen; SaO_2_: arterial oxygen saturation; SD: standard deviation; VO_2_: global oxygen consumption.

## Competing interests

The authors declare that they have no competing interests.

## Authors' contributions

AP conceived the study, ran the experiments, performed the analysis and wrote the manuscript. FF measured mitochondrial enzyme activities and participated in data analysis. MM ran the experiments. SV participated in data analysis and helped to draft the manuscript. SG participated in the study design and ran the experiments. GPC participated in the study design and analysis. RDG measured markers of oxidative stress. LG participated in the study design and helped to draft the manuscript. All authors read and approved the final version of the manuscript.

## Supplementary Material

Additional file 1 **Hemodynamic changes during metformin infusion**. Ten pigs were infused with metformin and five others were not. Changes in heart rate, mean arterial pressure, cardiac output, diuresis, saline and norepinephrine infusion are reported. Click here for file

Additional file 2 **Hemodynamic changes during metformin or lactic acid infusion**. Ten pigs were infused with metformin and five others with lactic acid. Changes in heart rate, mean arterial pressure, cardiac output, diuresis, saline and norepinephrine infusion are reported. Click here for file
