# Supplementary material for: Metformin overdose, but not lactic acidosis per se, inhibits oxygen consumption in pigs
Source: Crit Care. 2012 May 8;16(3):R75. doi: 10.1186/cc11332 (PMC3580617; doi:10.1186/cc11332)

**Additional file 2.** Hemodynamic changes during metformin or lactic acid infusion. Ten pigs were infused with metformin (black bars) and five pigs with lactic acid (white dotted bars). Heart rate (**A**), mean arterial pressure (**B**), cardiac output (**C**), diuresis (**D**), saline (**E**) and norepinephrine (**F**) infusion were recorded every hour. Data are reported as mean and SD. * p<0.05 *vs.* time 0 within group.


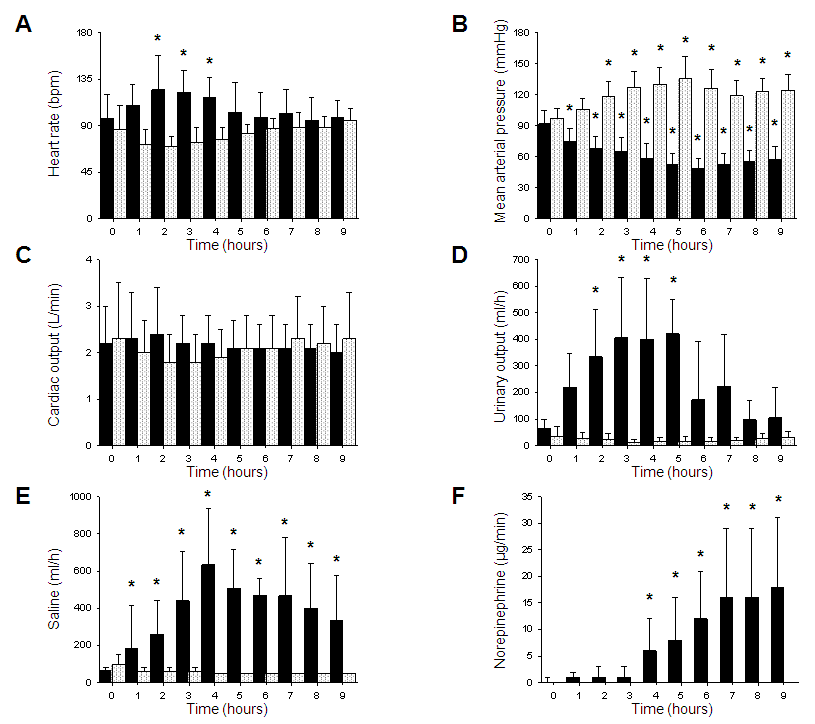

Supplement: Additional file 2 — Hemodynamic changes during metformin or lactic acid infusion. Ten pigs were infused with metformin and five others with lactic acid. Changes in heart rate, mean arterial pressure, cardiac output, diuresis, saline and norepinephrine infusion are reported. [file cc11332-S2.DOC]
